# Medical Society of the State of New York

**Published:** 1862-03

**Authors:** 


### MEDICAL SOCIETY OF THE STATE OF NEW YORK.

### FIFTY-FIFTH ANNUAL SESSION.

The Society met in Albany pursuant to statute, at 11 A. M., on Tuesday, February 4, 1862, for its Fifty-fifth Annual Session.

The President, *Dr. E. H. Parker* of Poughkeepsie, in assuming the duties of the chair, made a few brief remarks. After alluding in an appropriate manner to the death of his immediate predecessor in office, he called attention to the unusual prominence which military surgery had attained since the meeting of the Society, and urged upon all present to cultivate a knowledge of that particular branch, in order to be prepared, if required, to give their services to their country. He also mentioned the fact, that the State of New York was the first which had taken decided steps against admitting into the ranks of the volunteer corps any but competent surgeons; and in consideration of such a step, hoped that the Society would express their approval of it by a suitable vote of thanks.—Referring to the duties of the military surgeon upon the battle-field, he spoke of the necessity of the wounded who were left behind being properly cared for by their own surgeons. Alluding to the heroism of those surgeons who remained with their wounded after the battle of Bull Run, he suggested that the Society should take such action as should show these gentlemen, whether from this or other States, that their heroism was noticed by the profession. He had also hoped to be able to propose some plan, by the adoption of which the sufferings of war might, so far as the wounded were concerned, be alleviated by allowing the surgeons of both armies to visit the battle-field for this purpose, but had met so many obstacles that he had desisted from the attempt. He, however, urged the Society, if they could devise any method of accomplishing this, to do so.

The following Committees were next announced:

*Committee on Credentials.*—Drs. Porter of Oneida, Ferguson of Warren, and Willard of Albany.

*Committee on Nominations.*—Drs. Bissell of Oneida, Vanderpoel of Albany, Crispell of Ulster, Bly of Monroe, Finnell of New York, French of Broome, Hall of Cayuga, Reynolds of Saratoga.

The Secretary, *Dr. S. D. Willard* of Albany, presented a memoir of Dr. Merrit H. Cash of Orange, and announced that $500 had been bequeathed to the Society by the deceased member.

*Dr. Bowen* of Oswego offered the following:

*Resolved*, That a Committee of two be named by the Chair, to extend an invitation to such members of the Legislature as belong to the medical profession, to attend the meetings of this Society during its present session.

The Committee consisted of Drs. Bowen and Potter.

*Dr. Bissell* of Utica also offered the following resolution, which was accepted:

*Resolved*, That the thanks of this Society be tendered to the President for his interesting and suggestive address, and that a copy of it be requested for publication in the Transactions of the Society.

*Resolved*, That a Committee of three be appointed, to consider and report such action thereon as may be deemed necessary.

Drs. Bissell, Townsend and Kendell, were chosen as that Committee.

PATHOLOGICAL SPECIMENS.—*Dr. T. C. Finnell* of New York, presented three pathological specimens. The first was the bones of the foot and leg, removed from a patient, æt. eighteen years, who eight years previous to her death suffered a compound fracture of the ankle-joint by the falling of a heavy piece of timber upon it. The foot was turned strongly inwards, and remained ever after in that position. The ankle-joint, tarsus, and metatarso-phalangeal articulations were firmly anchylosed. Interstitial absorption of the bones of the tarsus had taken place to such an extent as to leave a mere shell of the form of the bones. The second specimen was a portion of skull showing a compound fracture, caused by a blow from a brick, the interesting feature of the case being the presence of a portion of the missile firmly imbedded in the bony structure; this remained in that situation for two weeks previous to the death, without giving rise to any head symptoms. The third specimen was the skeleton of a monster. He also exhibited some barbarous-looking obstetrical instruments which had been the property of an old Cuban physician, consisting of pelvimeters, sounds, crotchets, forceps, perforators, etc. Lastly he read a case of dysentery in a child, æt. 9, ending in suppurative peritonitis, spontaneous perforation at umbilicus, with discharge of four pounds of pus, and perfect recovery at the end of four months.

The following communication, relative to the appointment of homœopathic surgeons to the army, was received from the Oneida County Medical Society:

*Whereas*, great exertions are now being made by circulating petitions throughout the country asking Congress to pass a law appointing homœopathic practitioners to the post of Army Surgeons, therefore

*Resolved*, That a Committee of three be appointed to memorialize the State Medical Society to take such measures as its wisdom may dictate to maintain the honor and position of the medical profession, and also to express to our member in Congress the decided disapprobation by this Society of such an unwise innovation.

On motion of Dr. White of New York, the communication was laid on the table, until a Committee from the Academy of Medicine should have an opportunity to present a series of resolutions having a similar import.

The Society then, on motion of Dr. Griscom, adjourned to meet at 3 P. M.

*Tuesday Afternoon.*

The Society was called to order at 3 P. M.

PARALYSIS AFTER DIPTHERIA.—*Dr. Bissell* of Utica, read a paper on "Reflex Paraplegia, as a sequel to Diphtheria." He supported the idea that the paralysis was the result of an altered nutrition in the periphery of the senitent nerves, affecting secondarily the spinal cord by reflex action. The amount of fatality of the cases which had come under his observation, was about five in nine. The treatment consisted of tonics and stimulants. A dorsal decubitus was strongly insisted upon, in order to allow the blood to gravitate to the spinal cord; and the indications in this particular were further carried out by dry frictions, warm flannels, and the internal use of strychnine, etc.

*Dr. Curry* of Westchester, was surprised at the average mortality in Dr. Bissell's cases. He had never met with a fatal case in his own pract'ce, and he was himself an example of a cure of the disease, after having suffered from it for a period of three or four weeks. Rather accidentally he found that a relief from, and in fact a total disappearance of the unpleasant symptoms could be had by exposure to cold, and the administration of a moderate amount of whiskey. He agreed entirely with the views expressed by Dr. Bissell in regard to the character of the affection.

*Dr. French* of Rome, had seen several cases of the disease under discussion, but they had all recovered.

*Dr. Marsh* of Onondaga, had met with four cases of paralysis. Two of these terminated fatally. He had frequently met with convalescent cases of diphtheria attended with slight amaurosis, and very many that suffered from paralysis of the bladder, as shown by the retention of urine.

*Dr. Govan* of Rockland County, stated that out of a large number of cases of diphtheria reported from Rockland County, he did not recollect a single case of parapl[illegible]gia.

*Dr. D'Avignon* of Clinton County, thought that the paralysis of the velum was dependent in a great measure upon the length of time that the deposit remained upon it.

*Dr. Curwen* of Wayne County, had seen but two cases of paralysis, and they both died.

*Dr. Taylor* of Onondaga County, had not known of a case of paraplegia occurring in his County, notwithstanding diphtheria had been very prevalent.

*Dr. Garrish* of New York, stated that he had frequently met with cases of paralysis similar to those referred to by the other gentlemen, and had found that stimulating frictions, in conjunction with tonics, were attended with the best of results.

Function of the Larnyx.—*Dr. Porter* on behalf of Dr. Boulware of Albany, presented a specimen of wound of the throat, through the cricothyroidean membrane, followed by occlusion of the breathing tube at that point. The person from whom the specimen was removed, was a female, æt. 23, who, during an attack of temporary insanity, endeavored to destroy life with a razor. The cut was not an extensive one, and no vessels of large size were wounded. The lips of the wound were brought together, and in the course of three weeks the parts had all healed with the exception of a small opening in the trachea, just below the cricoid cartillage.—An attempt was made to close this opening, but very soon mucus collected in the tubes in such quantities as to render the removal of the dressings necessary in order to prevent suffocation. The tracheal tube was then introduced into the opening, and, with but slight intermission, was worn for several weeks. At the end of about eleven weeks from the infliction of the injury, the silver tube having been for some time removed, the wound closed, but it was found necessary on account of the collection of mucus in the trachea to open it again. No further attempt was then made to heal the wound, and it was found that she began to lose her voice, the aphonia being complete in the course of a few weeks. At this time, by closing the opening in the trachea, breathing would be stopped, proving that air could not pass through the larynx. Notwithstanding she had no voice, she could make herself intelligible by whisper sounds. She eventually died nineteen months after the receipt of the wound in consequence of suffocation, induced by the collection of quantities of mucus in her breathing tubes.—The case was one which illustrated, in quite a satisfactory manner, the important and essential part which the larynx plays in the formation of the voice, while, at the same time, it proved that the laryngeal voice is not essential to the formation of whispers.

On motion, the Society then adjourned to meet on Wednesday, 10 A. M

*Wednesday Morning, Feb.* 5 1862.

After the reading and approval of the minutes of the previous meeting, Dr. O. White, of New York, called up from the table the resolution offered by the Oneida Co. Medical Society, relative to the appointment of homœopathic surgeons to the army.

*Dr. Garrish* then presented the resolutions adopted by the New York Academy of Medicine upon the same subject, which have been already published in the *Times*. Some discussion took place in reference to the adoption of the resolutions as the sense of the Society, and finally on motion of Dr. Coates, it was resolved that a committee of three be appointed to take into consideration the subject, and report at the next meeting.

The committee was constituted of Drs. Coates, Townsend and Squibb.

*Dr. E. R. Squibb*, of Brooklyn, in the absence of F. H. Hamiltôn, chairman, read an elaborate report from the special committee upon the United States Drug Law, with a brief history of the movement subsequent to the adjournment of said committee. He also read a report of his own duties as the representative of the Society in the committee of revision and publication of the U. S. Pharmacopœia.

*Dr. S. D. Willard* presented a paper entitled, Conservative Surgery, with a list of surgeons and assistant-surgeons of the volunteer army of New York, their age, where graduated, what year, what service seen, when appointed and where promoted.

MEDICAL PROVISIONS FOR RAILROADS.—*Dr. Edmund Arnold,* of Yonkers, next read an interesting, elaborate and practical paper "on the medical provision for railroads, as a humanitarian measure, as well as a source of economy to companies." After citing cases of various classes, and showing the loss of life arising from the neglect of such previous provisions, he detailed his plans for supplying it, much of which we have already given to our readers. As on many lines, however, stations and flag-stations are far apart and appliances would be too far off, he also detailed provisions to be carried in the cars themselves. Within the last few days Dr. Arnold had heard that a measure had actually been prepared to go before the Legislature with the consent of most of the railroad companies of the State, of which the medical provision forms an essential feature, and of which we may give an abstract in a future number.

*Dr. Mason*, of Kings Co., presented the following:—

*Whereas*, On the principle of self preservation being the first law of nature, it is the paramount duty of the State to promote by all possible means the preservation of the health and lives of the people, and their protection against the causes of disease which continually surround them, especially in connection with the conditions of civilization and whereas, in the opinion of this Society, the health laws of this State have not kept pace with the rapid modern progress of sanitary science and government fails to enforce many well known means by which disease and death may be averted and longevity and population increased, therefore

*Resolved*, That the bill before the Legislature, known as the Metropolitan Health Bill, meets with the cordial approval of the State Medical Society, as a measure which, though partial in its application to one section of the State, is a step in the right direction and should be enacted into a law without delay.

*Resolved*, That the foregoing preamble and resolution be authenticated by the officers of this Society and transmitted to the two Houses of Legislature

The resolutions were warmly supported by Drs. Griscom, Mason, Taylor and others and were finally adopted.

*Dr. Hutchinson*, of Kings Co., read a paper on "Dislocation into the Ischiatic Notch, with Autopsy," which illustrated the practicability of Reid's method of reduction.

*Dr. Downs*, of New York, followed with the synopsis of a case of peritonitis, occurring in a child, in which large doses of morphine were used in the treatment.

*Dr. Brinsmade*, of Troy, offered the following resolution, which was adopted:

*Resolved*, That a Committee of five be appointed to draft a Sanitary Code for the State of New York, and submit the same to this Society for its consideration, at its next annual meeeing.

Drs. Brinsmade, Seymour, Griscom, Hun and Mason, were appointed on the said Committee.

Cirrhosis of Liver—Vomiting of Blood.—*Dr. M. M. Marsh*, of Onondaga, presented a specimen of cirrhosis of the liver. The patient vomited during eighteen hours more than nine pints of blood, and after the lapse of four days again vomited eighteen and a half ounces of the same fluid. Autopsy revealed a softened condition of the vessels of the duodenum, "hob-nailed" liver, and enlarged spleen. The spleen was double, each separate organ being supplied by a branch of the splenic artery, and being made perfectly distinct from each other by a membrane between; one was directly over the other.

*Dr. Finnell* stated that in over one thousand post-mortem examinations made by him, he had not met with a condition of the spleen similar to that described by Dr. Marsh, where the organs were placed in such relation to each other with a membrane intervening. He had, however, not uncommonly seen a series of spleens in the same subject, each supplied by a distinct arterial twig. In reference to the cause of death, he could call to mind two cases presented by him to the N. Y. Pathological Society, where death from hæmatemesis was simply the result of cirrhosis of the liver.—Both these patients were young females. He also stated that the amount of blood lost in the case was an interesting fact to note.

*Dr. Hart*, of Brooklyn, having frequently had occasion to notice the concurrence of enlarged spleen with cirrhosis, asked if such was always the case.

*Dr. Finnell* replied in the negative.

*Dr. Garrish* stated that he had frequently met with a normal spleen in cirrhosis.

The Society then adjourned to meet at 3 P. M.

*Wednesday Afternoon.*

The minutes of the morning were read and approved.

*Dr. Van Hœvenburgh*, of Ulster, read the following:

*Resolved*, That a Committee of five be appointed by the Chair to see Dr. Freer, chairman of the Senate Committee on Medical Societies, as also the Medical Committee of the House, and inquire the provisions of the bill incorporating the State Homœopathic Medical Society, and report to this Society, what action is necessary in the premises.

Adopted, and Drs. Vanderpoel, Bissell, Blatchford, Bates and Taylor, were appointed such Committee.

*Dr. Shrady*, of New York, offered a preamble and resolution relative to the medical provision for railroads, as advocated in the paper read by Dr. Arnold during the morning session:

*Whereas*, In the opinion of this Society, much loss of life and limb occur for want of sufficiently speedy medical assistance in cases of railroad accidents, and whereas the efforts of medical men when present are often rendered nugatory by the want of suitable appliances, and whereas it is desirable that some better provision should be made than at present exists to prevent railroad casualities, and whereas this Society has been informed that a large and comprehensive measure is about to be introduced in the Legislature of this State, of which proper medical attendance for railroads forms an essential feature, therefore be it

*Resolved*, That a Committee be appointed to report at the earliest moment whether any or what action shall be taken by this Society, in the premises.

The Committee consisted of Drs. G. F. Shrady, E. Arnold and A. Willard.

*Dr. Harris*, of New York, as Chairman of a Committee on the Medical Topography of the State, sent a communication reporting progress.

*Dr. Blatchford* announced that the New Jersey State Medical Society desired the appointment of a delegate to the New York State Society, and moved that six delegates be named to attend the next annual meeting of that Society, which would be held in Jersey city.

CASE OF SUPPOSED MURDER.—*Dr. John Swinburne*, of Albany, read an elaborate paper treating of the medico-legal points in the celebrated Budge case. He gave at great length his reasons for supposing it to be a case of murder instead of suicide, and in conclusion read corroborative letters from Geoghehan, Taylor Mott, Gross and others.

*Dr. Squibb*, from the Committee to which was referred the resolutions of the Oneida County Medical Society and the New York Academy of Medicine, upon the subject of remonstrating against the introduction of Homœopathy into the Army, reported that the Society should earnestly endorse the object of these resolutions, but advised that all unnecessary action that might be construed into persecution be avoided; that the committee felt satisfied that the Government will take no step so disasterous, so revolutionary and so expensive, as the one of introducing any forms of charlatanry into the Army.

*Dr. J. N. Minor*, of Kings Co., read a description of a new instrument for the treatement of stricture of the urethra, and Dr. J. A. Burge, Kings Co., followed with an account of important modifications made in the instrument, for a similar purpose, presented by him at the last annual meeting.

The Society then adjourned to meet in the Assembly Chamber, at 7½ P. M., to listen to the President's address,

*Wednesday,—Evening Session.*

The meeting being called to order by the Secretary, the President Dr. E. H. Parker, delivered his annual address. He chose for his subject, the dignity of the profession, and discoursed upon the ennobling and distinctive qualifications of the physician. The whole was treated of in an exceedingly happy manner and called forth from the assembled audience the most profound attention. We regret our inability at present to give an abstract of his remarks, but hope to do so on a future occasion.

*Dr. Kendall* presented a resolution of thanks for the address, accompanied with a request for its publication. The Society then adjourued to meet at 9 A. M., on Thursday. The further entertainment of the evening was left to Surgeon-General Vanderpoel and Dr. Swinburne, who received the members in turn at their respective residences.

*Thursday,—Morning Session.*

The Society was called to order by the President. E. H. Parker, and the minutes were read and approved.

*Dr Jas. V. Kendall,* as Chairman of the Committee on the introductory address of the President, made a report in a series of resolutions, complimentary to the Surgeons at Bull Run, and those who would not except parole.

He also presented the following:

*Whereas* An inscrutable but all-wise Providence has seen fit since the adjournment of our last annual meeting to remove from our midst by death, one of our members, the late President of the Society, therefore,

*Resolved,* That in the decease of Dr. Daniel T. Jones, the members of the Society are solemnly taught the scriptural lesson, that "man's breath is in his nostrils," that life, health and all their concomitant blessings, are dependant upon the will of our Supreme Ruler; and that it becomes us to bow submissively to his will, and have our work done, like our deceased member, for our call when the Master shall come.

*Resolved,* That in the decease of our brother, this Society has lost one of its most earnest, efficient and valuable members; his patrons one of the safest and most judicious of medical advisers; his family the best of husbands and fathers, and the community in which he lived, a generous, noble, upright, honest man; and that his virtues as member of different communities, but of the same medical Society, it becomes us to imitate.

Officers for the Ensuing Year.—The Committee on nominations then made the following report, which was adopted: For *President,* Thomas Hun, of Albany; *Vice-President,* D. P. Bissell, of Utica; *Secretary,* S. D. Willard, of Albany; *Treasurer,* J. V. P. Quackenbush, of Albany; *Committee on Publications,* Thomas Hun, S. D. Willard and Howard Townsend; for *Censors, Southern District,* W. Govan, Joel Foster and E. Harris; *Eastern District,* B. F. Staats, J. W. Blatchford and P. McNaughton; *Middle District,* J. S. Sprague, C. B. Coventry and A. P. Doolittle; *Western District,* Alex. Thompson, H. W. Dean and E. Hall.

*Dr. Shrady,* as Chairman of the Committee to report on the medical provision for railroads, offered the following for adoption:

*Whereas,* This Society has heard that a measure is about being introduced into the Senate, of which an essential feature is thorough medical provisions for railroads, and whereas we believe that much loss of life and limb, results from want of such provision, therefore,

*Resolved,* That we hail with satisfacion the introduction of any plans calculated to secure so desirable an end.

*Resolved,* That a copy of the foregoing be forwarded to Senator Smith, of Kins

Co., the gentleman who had given notice to the Senate, of the introdaction of such a measure.

The following papers were next read: By Dr. Burge, of Kings County, "A new instrument for removing foreign bodies from trachea and œsophagus;" Dr. Bly, Rochester, "On proper points for amputation."

*Dr. Quackenbush*, of Albany, in behalf of Dr. Van Dyck, exhibited a specimen of monstrosity, and remarked upon a peculiarity which existed in its formation, viz.: that the thoracic and abdominal viscera were external to the body. The cord was little over two inches in length. The monster was the product of an abortion in a young unmarried female.

*Dr Lee*, Peekskill, gave a description of a new field tourniquet, devised by Dr. Lambert.

*Dr Swinburne*, exhibited a patient upon whom he had some time since performed the operation of exsection of the hip-joint.

The following resolutions were in turn offered and adopted.

*Dr. White*, New York:

*Resolved*, That the thanks of the State Medical Society be accorded to its Secretary, Dr. S. D. Willard, for the laborious compilation he has made of the names of the medical men who have entered the Army from the State of New York.

*Dr. Lee*, Peekskill:

*Resolved*, That a Committee of three be appointed to compare the code of ethics adopted by this Society, in 1833, with that of the American Medical Association and present the revised copy to the Secretary at the next annual meeting.

Drs. Lee, Minor and Townsend, were appointed.

*Dr. Coates*:

*Whereas*, It becomes us as dependant upon the all-wise Being, for guidence in all our transactions, therefore,

*Resolved*, That hereafter the proceedings of our annual meeting be inaugurated with prayer, and that the Secretary be requested to invite the attendance of some clergyman to act as chaplain.

After the passage of a vote of thanks to Drs. Oakely, Vanderpoel and J. Swinburne, the Society adjourned *sine die.—N. Y. Medical Times.*

---

PHTHISIS.—In a recent communication to the French Academy of Medicine, by M. Piorry, on the treatment of phthisis, he presents the following summary of conclusions:—

1st. Pulmonary phthisis is a collection of numerous morbid phenomena, and not a morbid unit.

2d. There does not exist, nor can there be, a special remedy for it, to destroy a unit which has no existance.

3d. That consequently iodine, tincture of iodine, no more than chlorine, salt, tar, can be considered as anti-phthisical.

4th. That it is necessary, in order to the proper treatment of phthisical persons, to appreciate and specify the particular organic affections which they present, and to meet them with appropriate remedies.

5th. That hygienic precautions, intelligently advised, may prevent the development of tubercle.

6th. That by proceeding in this way, by combating the particular affections which occur together or succeed each other, we have a rational treatment of phthisis, which can show a fair number of perfect cures, and a very large number of palliated cases.—*Chicago Med. Ex.*

